# The CAMH Neuroinformatics Platform: A Hospital-Focused Brain-CODE Implementation

**DOI:** 10.3389/fninf.2018.00077

**Published:** 2018-11-06

**Authors:** David J. Rotenberg, Qing Chang, Natalia Potapova, Andy Wang, Marcia Hon, Marcos Sanches, Nikola Bogetic, Nathan Frias, Tommy Liu, Brendan Behan, Rachad El-Badrawi, Stephen C. Strother, Susan G. Evans, Jordan Mikkelsen, Tom Gee, Fan Dong, Stephen R. Arnott, Shuai Laing, Moyez Dharsee, Anthony L. Vaccarino, Mojib Javadi, Kenneth R. Evans, Damian Jankowicz

**Affiliations:** ^1^Krembil Center for Neuroinformatics, Center for Addiction and Mental Health (CAMH), Toronto, ON, Canada; ^2^Dalla Lana School of Public Health, Toronto, ON, Canada; ^3^Business Intelligence, Center for Addiction and Mental Health (CAMH), Toronto, ON, Canada; ^4^Ontario Brain Institute, Toronto, ON, Canada; ^5^Indoc Research, Toronto, ON, Canada; ^6^Rotman Research Institute, Toronto, ON, Canada; ^7^Department of Medical Biophysics, University of Toronto, Toronto, ON, Canada

**Keywords:** neuroinformatics, collaborative brain science, medical informatics, XNAT, LabKey

## Abstract

Investigations of mental illness have been enriched by the advent and maturation of neuroimaging technologies and the rapid pace and increased affordability of molecular sequencing techniques, however, the increased volume, variety and velocity of research data, presents a considerable technical and analytic challenge to curate, federate and interpret. Aggregation of high-dimensional datasets across brain disorders can increase sample sizes and may help identify underlying causes of brain dysfunction, however, additional barriers exist for effective data harmonization and integration for their combined use in research. To help realize the potential of multi-modal data integration for the study of mental illness, the Centre for Addiction and Mental Health (CAMH) constructed a centralized data capture, visualization and analytics environment—the *CAMH Neuroinformatics Platform—*based on the Ontario Brain Institute (OBI) Brain-CODE architecture, towards the curation of a standardized, consolidated psychiatric hospital-wide research dataset, directly coupled to high performance computing resources.

## Introduction

Mental illness affects one in three individuals in their lifetimes (Smetanin et al., 2011), and is the leading cause of disability in Canada (Lim et al., 2008; Mental Health Commission of Canada, 2014; Whiteford et al., 2015) exerting an economic burden estimated at 51 billion per year, including health care costs, lost productivity and reductions in health-related quality of life (Lim et al., 2008; Smetanin et al., 2011). Investigations of mental illness have been enriched by the advent and maturation of neuroimaging technologies and the rapid pace and increased affordability of molecular sequencing techniques (Lynch, 2003; Linden, 2012; Factors Study, 2013; Fu and Costafreda, 2013; Schreiber et al., 2013; Mayberg, 2014; Etkin, 2014; Power et al., 2016; Altman et al., 2016).

While these tools can independently provide powerful insights into the brain’s structure and function, directed integration of complementary information holds considerable promise to accelerate discovery and identify cross-modal biomarkers for stratification, diagnosis and treatment of mental illness (Potkin et al., 2014; Mufford et al., 2017).

This increased volume, variety and velocity (Bellazzi, 2014; Lee and Yoon, 2017) of research data, presents a considerable technical and analytic challenge to curate, federate and interpret, requiring the adoption of clear standardizations and aligned infrastructure to coordinate data within and across studies. Neuroinformatics has emerged as a discipline in response to these needs and the progressive evolution of computational psychiatry.

To help realize the potential of multi-modal data towards the study of mental illness, the Center for Addiction and Mental Health (CAMH) constructed a centralized data capture, visualization and analytics environment—the *CAMH Neuroinformatics Platform—*based on the Ontario Brain Institute’s (OBI) Brain-CODE platform, enabling the curation of a standardized, consolidated psychiatric hospital-wide research dataset, directly connected to high performance computing resources.

The CAMH Neuroinformatics platform was developed to support core capabilities for institutional researchers:

- Provide a research data management platform that can accommodate and federate the varied research data collected at an academic teaching hospital.
- Provide value to researchers through data visualization, quality reports and intuitive query interfaces.
- Accelerate analytics, by bringing organized data structures and compute power together in an integrated environment.
- Establish a standardized framework, to facilitate cross-institutional data integration.

This article centers on the recent implementation of the CAMH Neuroinformatics Platform, a hospital-focused adoption of the OBI’s Brain-CODE model to enable organization of site-wide multi-modal research data to accelerate discovery in mental health. The manuscript addresses the utility and flexibility of Brain-CODE as applied to a hospital environment, and the extensibility of the model, as demonstrated by further developments, including the federation of anonymized clinical records and coupling to unified compute resources.

## Materials and Methods

To develop a centralized data management and analytics environment, CAMH approached the OBI to review the design elements of the Brain-CODE platform for large-scale multi-dimensional provincial data management, guided by the FAIR data principles (Jeanson et al., 2014, 2016; Wilkinson et al., 2016; Vaccarino et al., 2018). The Brain-CODE model met core criteria appropriate for translation to a research hospital environment.

Flexible
Brain-CODE adopted data capture and organization systems to support the vast array of data types found in brain science. This was essential to meet the requirements posed by the considerable variety of research data collected at CAMH, including magnetic resonance imaging (MRI), positron emission tomography (PET), computed tomography (CT), electroencephalography (EEG), genetics, epigenetics and proteomics. The systems were also extensible to adapt custom data types and structures. This flexibility extended through the choice of technologies, each of which allow for considerable customization, and open integration with other systems, including the addition of other databases, such as in the case of electronic medical record (eMR) datasets (CERNER), administrative data (such as the Institute for clinical evaluate sciences, ICES), population health and economics data.

Scalable
The Brain-CODE platform was demonstrated to be highly scalable as applied to province-wide neuroscience studies supported through the OBI. This scalability met the requirements to aggregate data across hospital research programs and to facilitate national and international multi-site studies. The platform needed to be capable of handling the hundreds of active studies CAMH supports and the thousands of closed/archived projects of historical data.

Secure
Brain-CODE was developed with a “privacy by design” approach, embedding security into each layer of implementation based on the 10 Canadian Standards Association (CSA) Privacy Principles^1^. This aligned with the requirements of a hospital environment, where security of research and clinical data are paramount. Granular and defined access levels, built around the structure of research endeavors, provided a solid framework for secure access.

Accessible
The individual applications and interfaces are highly accessible to the research community. The web-based tools are intuitive and well-suited for data collection in each domain (imaging, molecular, clinical), and require limited training to reach a sufficient level of comfort for systems adoption and can be made accessible securely within the hospital network, through centralized two-factor authentication.

### Research Domain Databases

**Figure 1:**
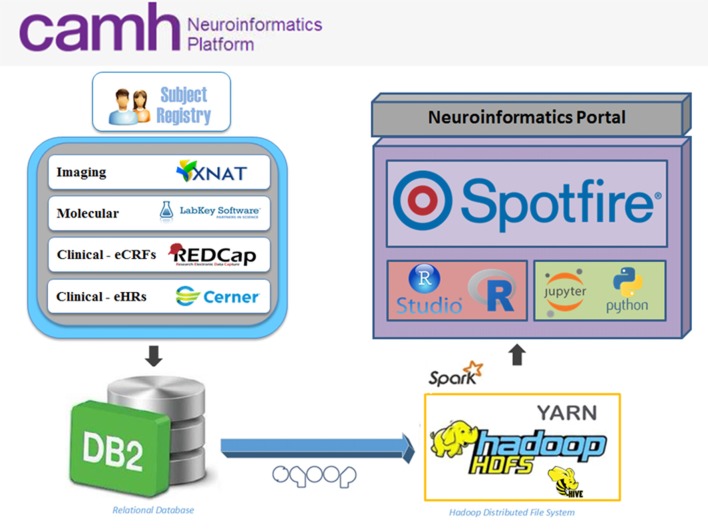
Overview of the Center for Addiction and Mental Health (CAMH) Neuroinformatics Platform. Data sources include XNAT (imaging), LabKey (molecular), REDCap (electronic case report forms, eCRFs) and case of electronic medical record (eMR) case of electronic medical record (eMR) datasets (CERNER; electronic health records, eHRs) which are federated into a central DB2 database. Federated datasets are available to compute resources (compute and Hadoop clusters) and easily accessible through dashboards and software notebooks through the Neuroinformatics Portal.

The Neuroinformatics Platform consists of open-source domain-specific database systems, federated through a DB2 back-end to provide subject-by-subject records. Each database interface is designed for a particular data-type, e.g., imaging, molecular, clinical, allowing for intuitive data entry and handling (Figure 1).

REDCap^2^ is used to capture behavioral and clinical assessments, including harmonized common data elements (CDEs) and self-report surveys (Harris et al., 2009). The CAMH instance of REDCap was validated in collaboration with the internal research ethics board (REB) and IT Security teams, to enable usage in regulated clinical trials in compliance with Health Canada.

XNAT^3^ (adapted as SPReD^4^) is used to store and organize medical imaging data, including MRI, CT/PET and EEG. MRI data are stored in both their original DICOM and derived formats, including NiFTI, MINC and ANALYZE, automatically generated through pre-processing pipelines.

LabKey^5^ is used for the coordination and storage of biological specimens and molecular data, including genetics, epigenetics and proteomics. This system supports both raw data storage and direct tabularization of results.

The databases support both original source data, derived values (e.g., quality assessments and final results) and pre-processed datasets (e.g., artifact correction).

All subject data are collected with informed consent, under a study-specific REB protocol. Authentication has been harmonized through the hospital-wide active directory system and within each sub-system, rights are limited depending on user-role to maintain security and to separate projects based on REB study protocol. All changes to user access require submission of an auditable electronic form, which requires principle investigator sign-off. This extends to visualization dashboards and individual table access for analytics (Clinical data access has additional constraints, described in the section specific to clinical record data).

In the current phase, external access can be provided to researchers who are named collaborators on the REB study protocol. Access requires confidentiality agreements and a centrally administered institutional account.

### Data Federation

Multi-modal datasets are federated using the IBM InfoSphere Federation Server^6^, which provides a thin, virtual data definition layer that allows seamless communication with data sources. A flexible API backend utilizes this federation capability to provide subject-oriented, de-normalized mart-like data tables, within a DB2 database environment. Data are linked, by unique standardized research participant IDs, across each source system, to generate a subject-level, profile for each individual.

### Visualization and Query Interface

Visualization and federated query interfaces are provided through TIBCO Spotfire^7^. Dynamic dashboards, refreshed daily, provide federated data views across data sources. These data views are served to specific research teams, defined by their study protocols and data requirements.

Dashboards provide visualizations that can be constructed from any data or metadata in the source systems (XNAT, REDCap, LabKey and CERNER). Filters can be applied directly through interactive selection, or a variable-by-variable query interface, to refine cohorts for data export to compute cluster environments or local processing centers.

Statistical packages included with the dashboard implementation allow for clustering, regression and stratification of datasets, presenting an initial layer of rapid exploration and visualization, prior to offloading to dedicated compute resources for further investigation.

### Neuroinformatics Portal

**Figure 2:**
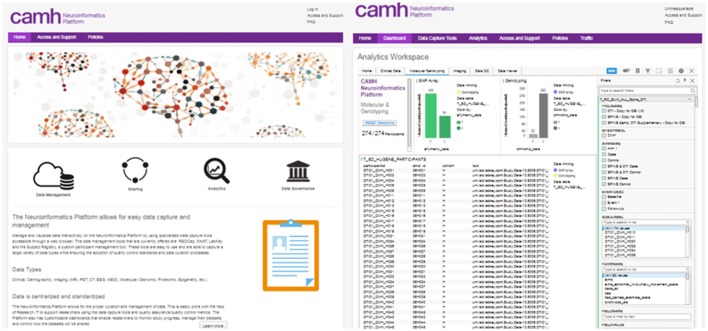
CAMH Neuroinformatics Portal landing page (Left), Dashboard view for multi-modal dataset (Right). The filter function for data query is illustrated for the Dashboard view.

Access to each of the data entry tools, dashboards and analytics applications are coordinated through a central *Neuroinformatics Portal* (Figure 2). This primarily web-based design of the Neuroinformatics Platform provides a consolidated gateway for CAMH researchers to interact with their data.

### Central Subject Registry

A central ledger of all participants entered into the platform is supported by the *Subject Registry* (Vaccarino et al., 2018). As a core component of this tool, medical record numbers (MRNs) or health card numbers can be encrypted on entry, allowing for the identification of common participants across studies. As participants can be identified across studies, visits and encounters, the subject registry facilitates longitudinal dataset linkages and simplified hospital-wide research participant review and oversight.

The Neuroinformatics Platform operates based upon informed participant consent, meaning that institutional REB approvals and associated informed consents govern what data can be collected, uploaded, de-identified and shared. This information is tracked in an Ethics Tracking Database, (supported through a validated REDCap instance) which contains information on the sensitivity of datasets and sharing permissions. The information in the Ethics Tracking Database is linked to each participant via the Subject Registry which allows the tracking and management of data permissions on a participant-by-participant basis.

### Quality Assurance

Prompt and reproducible metrics of data quality are essential to ensuring the integrity of research data. This is supported through the Neuroinformatics Platform in the implementation of quality control and quality assurance (QC/QA) scripts launched for new data entry into data collection systems, and the presentation of data quality dashboards.

QC scripts and summary dashboards are a core component of the XNAT implementation. Automated QC scripts are initiated on a nightly basis, with computation coordinated through the CAMH compute cluster. These include naming convention checkers, scan protocol checkers and both human and phantom QC/QA. Functional MRI data quality is assessed using phantom and human implementations of the fBIRN pipeline from the Biomedical Informatics Research Network (Friedman and Glover, 2006; Glover et al., 2012). Structural data, specifically T1 scans are evaluated through an MRI registration pipeline that automatically registers (non-linear warping with ANTS^8^ every new high-resolution T1 MRI structural scan to a template and then automatically measures signal-to-noise (SNR) and contrast-to-noise (CNR) in gray matter. The pipeline also includes white matter measures and automatically measures volumes of interest using the MNI152 registration template and the LPBA40 segmentation atlas (Shattuck et al., 2008).

**Figure 3:**
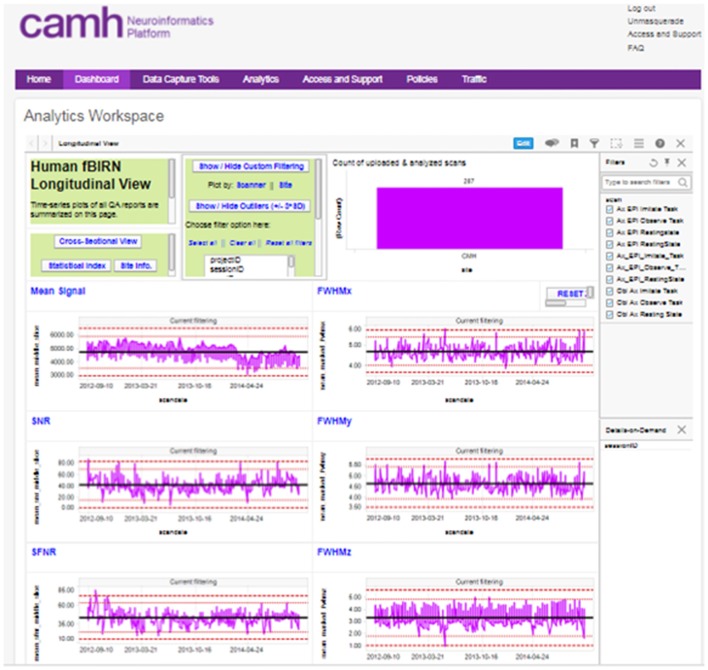
Example, “global” longitudinal quality assurance and quality control (QA/QC) dashboard for functional MRI (fMRI) data.

The reports generated by these scripts are captured and associated with the subject/imaging sessions in XNAT, and are further aggregated into interactive dashboards visible to each research group, with both cross-sectional and longitudinal views across the study (Figure 3).

A “global” imaging data quality dashboard also provides a full view of all data entered into the Neuroinformatics platform. This assists with the evaluation of overall site performance, long-term trending and detection of outlier data.

Any number of pipelines can be added to these workflows to support additional QC or pre-processing steps on neuroimaging datasets that can be executed on secure local compute resources.

#### XNAT—Anonymization

In additional to anonymization of clinical data discussed in the following sections, de-identification of imaging data is also handled through automated pipelines (Li, 2011). A DICOM header de-identification pipeline is applied to remove or replace fields within the DICOM files. The fields to be modified are configurable and are evaluated on a project-by-project basis, dependent on REB protocol and in co-ordination with the CAMH privacy office. High-resolution structural MRI scans have been demonstrated to allow for the reconstruction of facial features and identification of individuals (Schimke et al., 2011). To support anonymization of imaging data a defacing pipeline based on the MRI_deface tool (FreeSurfer; Bischoff-Grethe et al., 2007) can be applied to data to remove facial features from T1 images. In combination these pipelines can reduce the likelihood of re-identification of imaging datasets.

### Clinical Datasets

#### Electronic Medical Health Records

CAMH is a “HIMMS EMRAM Stage 7” hospital with highly coordinated electronic medical health records systems (CERNER) deployed to clinicians as I-CARE^9^. These records are of significant interest to researchers, both as independent sources of information related to patient prognosis, progression and outcomes, as well as when combined with research data, such as medical imaging and molecular expression.

Clinical datasets are provisioned to researchers through two methods: (1) anonymized aggregate data for review by internal researchers; and (2) data cuts specific to a REB approved study, including retrospective chart review, restricted only to those named members on the study protocol and in agreement with identifiers included when and if allowed by the REB.

**Figure 4:**
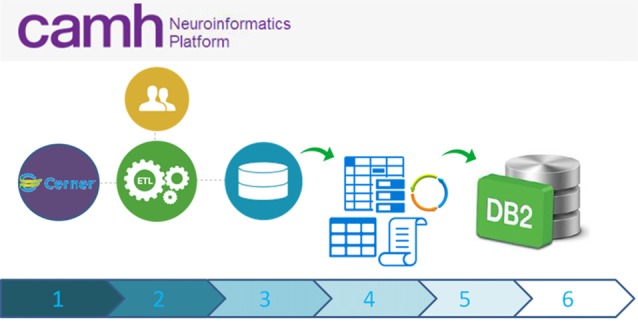
High-level schematic overview of data flow from the eMHR system (CERNER) to the Neuroinformatics Platform database: (1) Electronic Medical Health Care Data are collected as part of clinical care and from clinical trials/translational clinical research; (2) Extract Transform and Load (ETL) scripts extract data from the electronic medical health record system to a curated intermediary database; (3) The NI extraction scripts are run, pulling only the agreed upon variables and anonymous Research IDs. These data, including an up-to-date schema are transferred to a secure location; (4) Anonymization scripts (sdcMicro; Templ et al., 2015) are run to determine whether the new extract fulfills anonymization criteria. If not, data flow ceases and the data are triaged. The extract is revised, until the thresholds are appropriately met; (5) Once the anonymization thresholds are successful, data are transferred to the DB2 database, incorporating updated schemas; (6) Accesses to these data are provided securely to research teams, with prior research ethics approvals only.

Coordinated data extracts of the hospital electronic medical health record system, are staged through the federation server, and then imported into the DB2 data-lake (Figure 4). These records, including demographics, laboratory results and pharmacological information, are linked to extended research datasets, securely bridging clinical and research domains.

#### Anonymization

The capability to ensure anonymization is essential to the use of clinical data in a research environment. Three primary methods are applied to clinical data prior to exposure to research systems: direct identifier removal, k-anonymity and l-diversity (using the sdcMicro software package; Templ et al., 2015).

Direct identifiers, such as name, address, phone number, date of birth, as well as IDs (such as medical record and health card numbers) are isolated and removed. These variables are masked (i.e., cells are nullified or the columns are removed entirely from the table) in the standard extract for the Neuroinformatics Platform.

Anonymous “Research IDs,” following the CAMH research naming convention, are generated in-place of other internal IDs tied to identifiable information. The clinical team retains secure mappings, to recover information if re-identification is required.

Variables that pose an identification risk, alone or in combination with others, including Gender, Age Group, Local Health Integration Network (LHIN) and Major Program are considered Key Variables. To enforce k-anonymity (Samarati and Sweeney, 1998; El Emam et al., 2009) the datasets are processed for unique values or unique combinations of up to three variables, which if identified are nulled.

Confidentiality is breached if a set of subjects with the same combination of (up to 3) key variables has the same diagnosis. In these cases subjects have their key variables nulled, to enforce l-diversity, while guaranteeing a minimum loss of information (Machanavajjhala et al., 2007).

After the application of k-anonymity and l-diversity algorithms, risk measures related to the probability of identification are calculated, to help ensure low risk of disclosure and monitor the disclosure risk changes over time.

These metrics are calculated for each subject in two ways: (i) “Disclosure Risk” for a given subject is calculated as 1 divided by the number of subjects with the same combination of key variables. It will be 1 if the subject has a unique combination of key variables, considered unacceptable; and (ii) “Sample Frequency on Subsets,” is calculated using the Special Unique Detection Algorithm (SUDA2). A Data Intrusion Simulation (DIS) score is derived for each subject based on considerations of how unique the combination of key variables is (with higher weight for combination of fewer variables).

The output of this process is an anonymized dataset and a report that highlights the changes made to the original data and summaries of the risk measures of anonymity.

If the risk probability for re-identification exceeds established thresholds, further processing will cease and the data will remain in the staging area. The dataset is adjusted in coordination with clinical teams until the re-identification risk is reduced to within the set parameters.

#### Cohort Explorer

**Figure 5:**
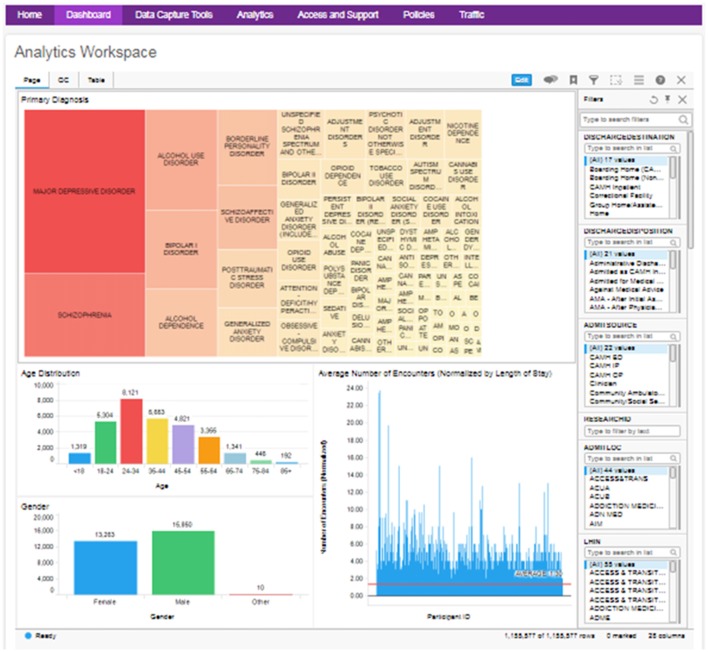
Example clinical data cohort explorer dashboard, with visualizations of diagnosis, age, gender and average encounter (filterable by diagnosis). QC data and full table views are also made available.

The anonymized medical record data are utilized to provide a cohort explorer for study feasibility evaluation and statistical power calculations (Figure 5). This follows a similar model to Informatics for Integrating Biology and the Bedside (i2b2; Murphy et al., 2006), by providing a layer of access to explore cohorts across the breadth of the clinical records systems. The clinical data can be further combined with research data from the other source databases through the common DB2 backend.

As the anonymization process can reduce the amount of information available, the aggregate cohort explorer is intended primarily as an overview to identify study feasibility. Further variables do continue to be added to the aggregate clinical extract, to make these data more valuable for analysis. Where further information is required, detailed extracts are provisioned in alignment with a specific REB protocol, and are anonymized as far as possible, to limit identifiers to those prescribed by the REB.

### Analytics

#### Compute Cluster

**Figure 6:**
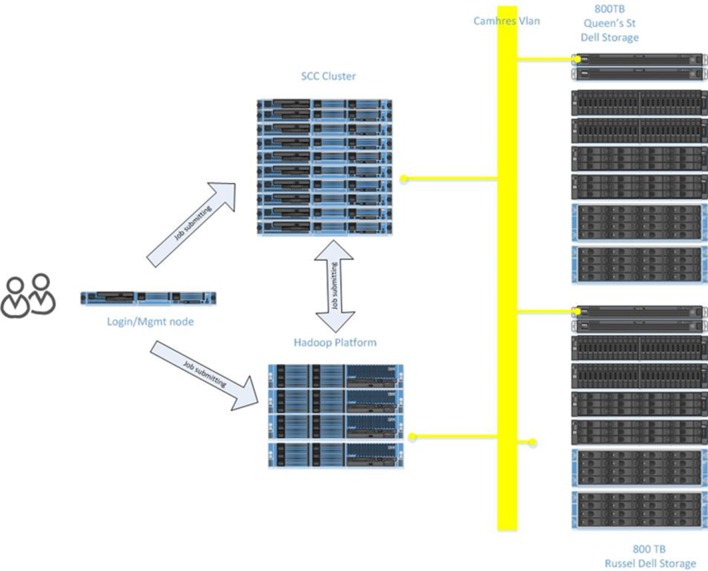
Illustration of the CAMH Compute Cluster architecture.

The scale and complexity of medical imaging and molecular datasets necessitates substantial compute capabilities for the pre-processing, QC measures and post-processing. The Neuroinformatics Platform was designed with full connectivity to a local high-performance compute cluster to handle computationally demanding tasks (Figure 6).

Automated scripts initiated from the source databases (e.g., XNAT and LabKey) are issued to the local compute infrastructure, on dedicated secure queues.

Researchers are able to access their datasets, via queries and data pointers directly from the compute clusters. The architecture adopted, minimizes data transfers, and includes a tightly connected network on a unified VLAN, at 10 GB bandwidth, between all Neuroinformatics platform resources.

#### Hadoop Analytics Environment

To enable analysis of increasingly large datasets, otherwise intractable to conventional approaches, the Neuroinformatics Platform was implemented alongside dedicated Hadoop infrastructure^10^. The DB2 database is imported in full to a *HIVE 2.0*^11^ framework, utilizing *SQOOP*^12^, with secured permissions enforced on a column-by-column level. Researcher’s datasets are directly accessible to the active workspace to apply pipelines and processing frameworks.

#### Notebook Interfaces

To further the accessibility and web-based design of Brain-CODE, notebooks for Python (Jupyter^13^) and R (RStudio^14^), common languages in computational psychiatry, are accessible through the central *Neuroinformatics Portal*. These notebooks can process code on either a classical compute cluster, or dedicated Hadoop environment, leveraging SparkR^15^ and PySpark^16^ to seamlessly execute pre-developed code, without recoding in native MapReduce.

#### Data Center

The infrastructure to support the functions of the Neuroinformatics Platform is maintained locally at CAMH across three secure data centers. The Neuroinformatics Platform adopted a design philosophy to ensure no “single point of failure.” Each server includes redundant components, network connections, RAID storage configurations and hot-spares.

Each database application (XNAT, LabKey, Spotfire and DB2) is provisioned with a dedicated development and production server, physically separated between the primary data centers for high availability and disaster recovery purposes.

**Figure 7:**
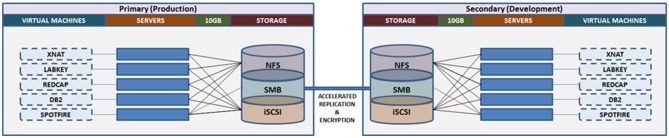
Overview of the Neuroinformatics Platform architecture that leverages high performance storage system replication and virtual machines, to support high availability, redundancy and robust failover.

Similar to the OBI, CAMH has adopted a primarily virtualized architecture, using Oracle VM (OVM^17^). While there are some limitations in performance as a result of virtualization, this approach provides substantial operational benefits, notably: (a) flexible deployment; (b) efficient snapshots for backup; and (c) simplified fail-over procedures to initialize replicated VMs. The virtual machines are distributed to a cluster of computers, through OVM, such that they can be dynamically deployed/re-deployed as required in case of hardware failure (Figure 7).

Data storage and backup functions are supported through a 1.9 PB high performance storage system. Replication at the file-level is conducted on an hourly basis, between the primary and secondary storage sites, maintaining concurrent mirrors of all raw and processed data (MRI, EEG, PET, etc.). Point-in-time snapshots are taken each day, and retained up to 1-month, such that accidental deletions or modifications can be rolled back for up to 30-days. Daily extracts of system configurations are included in the file-level replication.

The Neuroinformatics platform virtual machines are stored on a separate file system, accessed via Internet Small Computer Systems Interface (iSCSI), on the central storage system. This allows for block-level replication of the entire virtual machine environment between primary and secondary sites. Automated scripts allow for the preparation and launch of replicated virtual machines, (either the production or development frameworks), which can resume access of the research data from the file-level replica. Both replication channels are further accelerated using specialized hardware, and encrypted point-to-point.

The research storage systems, Neuroinformatics platform and high performance compute environments are interconnected by 10 GB optical fiber, under a single harmonized research VLAN. This interconnect provides high bandwidth and low latency to synchronize research data across applications and analytics systems. The compute infrastructure includes a Hadoop deployment (HortonWorks), a GPU node for machine learning applications, and 45 high memory (128–256 GB RAM) compute nodes, providing over 1,000 available processing cores.

This implementation of the Brain-CODE model on new hardware architecture demonstrates the flexibility of the design, and that it can be deployed under differing data center conditions.

## Results

The Neuroinformatics platform has provided a key component of technological infrastructure that affords researchers with a standardized framework for data organization and analytics, accessible through a centralized portal. The system, based on the OBI Brain-CODE framework, has been able to support and federate the varied research data types collected at CAMH.

At the time of writing, the CAMH Neuroinformatics Platform supports 38 distinct research projects, spanning each of the hospital’s primary research programs, with 3,61,777 total participant records (including medical records), and anticipated growth of 30,000 records per year (Table 1A). The total datasets span 20 TB and adoption across the hospital has been strong, with the web-based access model allowing for simplified study management and data transfer.

**Table 1:** Summary table of data currently stored in the Center for Addiction and Mental Health (CAMH) Neuroinformatics platform. **(A)** Neuroinformatics platform data summaries.

Primary database	Number of Participants
XNAT—Medical Imaging	2,878
REDCap—Assessments	13,514
LabKey—Molecular	15,385
eMHR—Clinical	330,000
**Total**	361,777
**(B)** Neuroimaging summary.
**Modality**	**Scans**
DTI	2277
EEG	1837
T1	2600
T2	4322
fMRI	22108
**Total**	33144

*Number of primary records stored in each database, XNAT, REDCap, LabKey and from clinical records, Summary of Neuroimaging data types currently stored in XNAT*.

Supported studies range multiple disorders and cross-lifespan populations including, Pediatric, Geriatric, Neurodegenerative (Alzheimer’s, Parkinson’s), Depression, Bipolar Disorder, Psychosis, Autism, Schizophrenia and Addictions (Alcohol, Nicotine). Data types include MRI: *Functional, Structural and Diffusion* (Table 1B), PET, EEG, Whole Genome Sequencing, Methylation, Chip Sequencing, MicroArray Sequencing and RNA Sequencing.

Each study varies in the data types that are required for collection and management. While not all studies include data across each domain (e.g., studies with molecular and assessment data, or imaging data only), several studies collect extensive phenotypic data incorporating medical imaging, molecular, assessment and clinical data for each participant.

In particular, the Social Processes Initiative in Neurobiology of the Schizophrenia(s) (SPINS^18^; *d* = 109) and Preventing Alzheimer’s Dementia With Cognitive Remediation Plus Transcranial Direct Current Stimulation in Mild Cognitive Impairment and Depression (PACt-MD^19^). These studies collect biological samples, neuroimaging data (with the inclusion of EEG data for PACt-MD) and extensive clinical and assessment data. The complex data collected by these studies are well supported by the CAMH Neuroinformatics platform as the system can accommodate the diverse data types and combine records through federation: SPINS (LabKey—274, REDCap—174, XNAT—319), PACt-MD (LabKey—230, REDCap—212, XNAT—217).

Tight coupling with computing environments supporting classic parallel clusters and Hadoop frameworks, avoids intermediary data transfer and storage, staging an environment for rapid data exploration at-scale. The analytics environments supporting the platform have run a total of 2,50,000 parallel jobs, spanning QC, pre and post-processing workloads. The use of web-based “notebook” interfaces has simplified access to computational resources and abstracted complexities of queue management from the user.

Federated records can be served securely to researchers through interactive dashboards, functionally refined to suit the requirements of each study. Dynamic query and filter functions embedded within the platform have enabled researchers to quickly identify cohorts and data sub-sets, greatly enhancing data accessibility, and shifting time spent on “collating data” to scientific interpretation.

The development of the Neuroinformatics platform establishes the first phase of hospital-wide data integration by providing a consistent framework for data organization and management.

## Discussion

Sophisticated systems are required to handle the increasing variety and scale of neuropsychiatric research data. These challenges are well-known to the neuroscience community, which have driven the development of several concurrent approaches to manage complex datasets including, FBIRN FIRE, COINS, LORIS, NeuroLOG, i2b2 and the Human Brain Project Medical Informatics Platform (Amorim et al., 2016).

### Comparisons to Similar Approaches

The Function Biomedical Informatics Research Network (FBRIN) and Federated Informatics Research Environment (FIRE; Keator et al., 2015) are a set of open-source integrated tools for multi-side or multi-study neuroimaging studies that includes many critical components such as central authentication, online clinical data entry forms and the Human Imaging Database^20^ for data management. FIRE also includes the FBIRN image processing stream^21^. This is a valuable open-source resource for functional MRI studies and shares several similarities with the CAMH deployment, including imaging and clinical assessment data collection, a centralized database and coupling to compute for processing pipelines (both including components of FBIRN QA). The two systems also share querying interfaces with URLs pointing to image data for staging downstream analyses. The Brain-CODE instance includes additional data sources, and has been extended for use with other neuroimaging data types, such as DTI.

The Collaborative Informatics and Neuroimaging Suite COINS^22^ (Scott et al., 2011) is based on an open-source model that includes web-based tools to manage studies, subjects, imaging, clinical data, and other assessments, including a standard metadata model and powerful query interface. It acts as an institutional data repository that enables secure data sharing with a focus on PHI considerations. While there are advantages to the COINS deployment, as compared to XNAT as a standalone implementation, such as longitudinal tracking and standardized meta-data and data structures, the Brain-CODE model incorporates strict standardization, including naming conventions for longitudinal studies and enhanced query through the federation system.

The Longitudinal Online Research and Imaging System (LORIS; Das et al., 2016) is an extensible web-based data management system that supports multiple data types, including imaging, clinical, behavior and genetics. The system includes capabilities to store, process and disseminate datasets and is used for a variety of multi-site studies with instances used worldwide.

It shares many conceptual components of Brain-CODE and the CAMH implementations, and provides valuable insight into the challenges of managing longitudinal research data. Compatibility between Brain-CODE and LORIS (Vaccarino et al., 2018) using the underlying federation model has been achieved to bridge these two systems towards data integration for specific studies.

NeuroLOG (Batrancourt et al., 2014) provides a middleware data management layer, to share heterogeneous and distributed neuroimaging data using a federated approach. Shared information can be captured through a multi-layer ontology and federation schema to harmonize heterogeneous data. This shares some components of the federation approach used in Brain-CODE, through standardization approaches and centralized federate schema. The challenge of combining retrospective heterogeneous datasets from legacy databases, still presents a challenge that may be addressed through the use of mappable data models and semantic database frameworks, discussed in relation to future work.

i2b2 is an open-source system developed to provide tools for clinical investigators to integrate medical records and clinical research data (Murphy et al., 2010). This provides similar functionality to the eMHR and research data integration provided through the CAMH instance of Brain-CODE, including a query tool to search applicable datasets, and are access restricted based on REB review. The i2b2 implementation also has two primary methods of exposure of medical record data: an anonymized dataset of researcher review and restricted matched sets of patients and controls based on study-specific requirements. The i2b2 platform uses ontologies to standardize data, and can link to diverse databases to access other data streams and connections to compute resources are supported. This system does lack the visualization capabilities afforded by Spotfire, and would rely on the source systems for QC.

The Human Brain Projects’ Medical Informatics Platform can provide support for hospital clinical data to be uploaded and maintained locally for analysis (without leaving the originating institution), and also view aggregated data for large-scale analyses of clinical data across hospitals (Galili et al., 2014). The CAMH Neuroinformatics platform approach is more similar to the i2b2 model, with data not yet federated in aggregate with other institutions. Secure aggregates are made available for internal use, however, the inclusion of data models and ontologies, coupled with anonymization, can allow for more broad clinical data integration.

In the context of the current environment of Neuroinformatics approaches, the Brain-CODE model as implemented at CAMH and its extension through local resources represents a unique application with several advantages suited to the hospital-focused use-case.

The Brain-CODE model utilizes open-source databases for imaging, molecular data and assessment data, leveraging the specialization of those tools to their data type(s). This supports a highly diverse range of modalities, as required by CAMH research programs. This also allows for new systems to be added, or replaced, as the Neuroinformatics field evolves. The underlying federation model has also been demonstrated to be flexible combining data from multiple internal and external data sources, such as eMHR data at CAMH.

The Neuroinformatics platform combines many of the key components of comparable systems, with flexibility to extend additional capabilities, to enrich the existing datasets and move towards institutional data integration.

### Limitations

There are several limitations to the implemented system, from a user perspective, repository perspective and the data federation approach.

Development of QC and pre-processing pipelines still requires substantial coding and subject matter expertise. Technical teams are available to assist researchers in implementing their pipelines under the existing frameworks (XNAT, LabKey), however, considerable knowledge of coding is still required to ensure that these analyses work seamlessly.

Work was done to allow for direct data download after querying federated study records. While this has been successfully implemented for imaging data from XNAT, the system can only provide tabularized molecular data from LabKey and has not yet been built to pull raw data in bulk through the query interface.

Many scripts and tools rely on standardized naming conventions for MRI scans, which have been shown to vary considerably between studies. While re-naming can be performed during data import, and look-up tables established to accommodate cases where re-naming is not possible, further effort is required to generalize the system to better handle varied conventions, particularly when considering inclusion of externals sources. The authors are also aware of the importance of provenance and maintaining full information about the sequence that was performed for data generation, which may preclude re-naming. Additional efforts are underway institutionally to standardize acquisitions.

As discussed in sections “Electronic Medical Health Records” and “Cohort Explorer” there are two methods that clinical data extracts can be made available: (a) as an anonymized aggregate; (b) a more complete extract dependent on REB approval for chart review. The anonymization framework for the clinical data is by design, conservative and results in a reduction of information available in the output records that make these data less useful to investigators. Ongoing efforts include adding additional variables to the aggregated extracts to provide further information of interest, while maintaining anonymization criteria.

A primary limitation of the current iteration of the Neuroinformatics platform is that while data are federated on a subject-by-subject level, they are not “integrated” across studies. These limitations exist for legal, ethical and technical reasons. Foremost patient consent and approved REB protocols are not generalized for data sharing. There are further technical limitations imposed by the initial federation software layer. It is a key component of current and future directions to implement an interoperability system, through Blue Brain Nexus^23^) supporting permutable data models and detailed provenance. Blue Brain Nexus was designed to fully support the FAIR data model, and is currently being implemented within the Neuroinformatics Platform to allow for findability, interoperability, accessibility and reproducibility. Through the development of standardized and consistent data model(s) that incorporate data sharing options and the technology of Nexus, will support the aggregation of different data sources for the purpose to increase study sample sizes and enrich a growing institutional dataset.

## Conclusion

The CAMH Neuroinformatics Platform represents a unique application of the Brain-CODE model in a hospital setting, enabling data management and federation between research and clinical domains, in support of treatment units and study centers.

The CAMH Neuroinformatics Platform supports individual study data management and lays the foundations to facilitate hospital-wide dataset federation, through the application of data standardization and CDEs^24^. Maximizing statistical power is challenging in individual studies, however, integration of related data through participatory consortia such as, ENIGMA (Kelly et al., 2018), ADNI (Yao et al., 2017), HCP (Van Essen et al., 2013), bioCADDIE (Cohen et al., 2017) demonstrate that more expansive datasets can be established for analysis. Thorough data integration requires the adoption of data models, ontologies and semantic description frameworks, to map between existing data and optimally coordinate future data collection and institutional developments of harmonized consent models. These capabilities are critical to the development of large-scale datasets from across diverse studies and the formulation of longitudinal datasets. The extensibility of the OBI Brain-CODE model allows these developments to be applied effectively at the individual domain-database level and the intermediary and federation layers.

Further expansion of the Neuroinformatics Platform will be focused on establishing a core integration layer that will ensure data remain “live,” in a searchable, accessible and interconnected format, under the FAIR data principles. Provenance will also be a cornerstone of future initiatives, embedded into the platform, to provide clear descriptors of data origins, processing pipelines and derivations, and to coordinate authorship in accordance with applicable data trajectories.

The implemented model of primarily open-source tools represents a crucial component of research infrastructure, which can be replicated at institutions of varying size to approach “Big Data” and multi-modal investigations. The Neuroinformatics Platform at CAMH will continue to accumulate multi-dimensional medical imaging, molecular and clinical data to further expand a rich dataset for large-scale studies to further our understanding of the etiology, progression and treatment of psychiatric illness.

## Author Contributions

Contributions to the development of the CAMH Neuroinformatics Platform: DR: led implementation of CAMH Neuroinformatics Platform. QC: administration for core research storage system and hardware. NP: REDCap development and integration. AW: CAMH cluster and compute administration, management of virtual infrastructure. MH: Neuroinformatics Platform administration support. MS: biostatistics support and clinical data anonymization. NB: cohort explorer clinical dashboard developer. NF: business intelligence lead for clinical data management. TL: data warehouse lead for clinical data extraction. BB: OBI project management support. RE-B: data federation development. SS: development of SPReD. SE: dashboard and visualization development. JM: molecular Data and Subject Registry support. TG: implementation project manager. FD: imaging database development. SA: quality control scripts and dashboard development. SL: imaging database development. MD: indoc lead for implementation. AV: clinical database development, common data elements. MJ: molecular database and dashboard development. DJ: institutional project lead. All authors have approved the manuscript and agree with submission to Frontiers in Neuroscience.

## Conflict of Interest Statement

The authors declare that the research was conducted in the absence of any commercial or financial relationships that could be construed as a potential conflict of interest.

## Footnotes

^1^https://www.csagroup.org/codes-standards/health-safety/

^2^http://project-redcap.org

^3^http://www.xnat.org

^4^https://sites.google.com/a/research.baycrest.org/informatics/spred

^5^https://www.labkey.com

^6^http://www-03.ibm.com/software/products/en/ibminfofedeserv

^7^http://spotfire.tibco.com/

^8^https://sourceforge.net/projects/advants/

^9^www.cerner.com

^10^http://hadoop.apache.org

^11^http://hive.apache.org/

^12^https://hortonworks.com/apache/sqoop/

^13^http://jupyter.org/

^14^https://www.rstudio.com/

^15^https://spark.apache.org/docs/latest/sparkr.html

^16^http://spark.apache.org/docs/2.1.0/api/python/pyspark.html

^17^http://www.oracle.com/technetwork/server-storage/virtualbox/overview/index.html

^18^http://camhstudies.ca/cgi-bin/ver2/findCAMHstudy_study.php?

^19^https://sunnybrook.ca/research/content/?page = sri-groups-nppc-proj-7

^20^www.nitrc.org/projects/hid

^21^http://www.nmr.mgh.harvard.edu/~greve/fbirn/fips/

^22^http://coins.mrn.org

^23^https://github.com/BlueBrain/nexus

^24^https://www.braincode.ca/content/getting-started#toc-2
